# Supplementary material for: RNA-seq and ChIP-seq Identification of Unique and Overlapping Targets of GLI Transcription Factors in Melanoma Cell Lines
Source: Cancers (Basel). 2022 Sep 19;14(18):4540. doi: 10.3390/cancers14184540 (PMC9497141; doi:10.3390/cancers14184540)
Supplement: Supplementary file 1 [file cancers-14-04540-s001.zip › Supplementary figures.pdf]

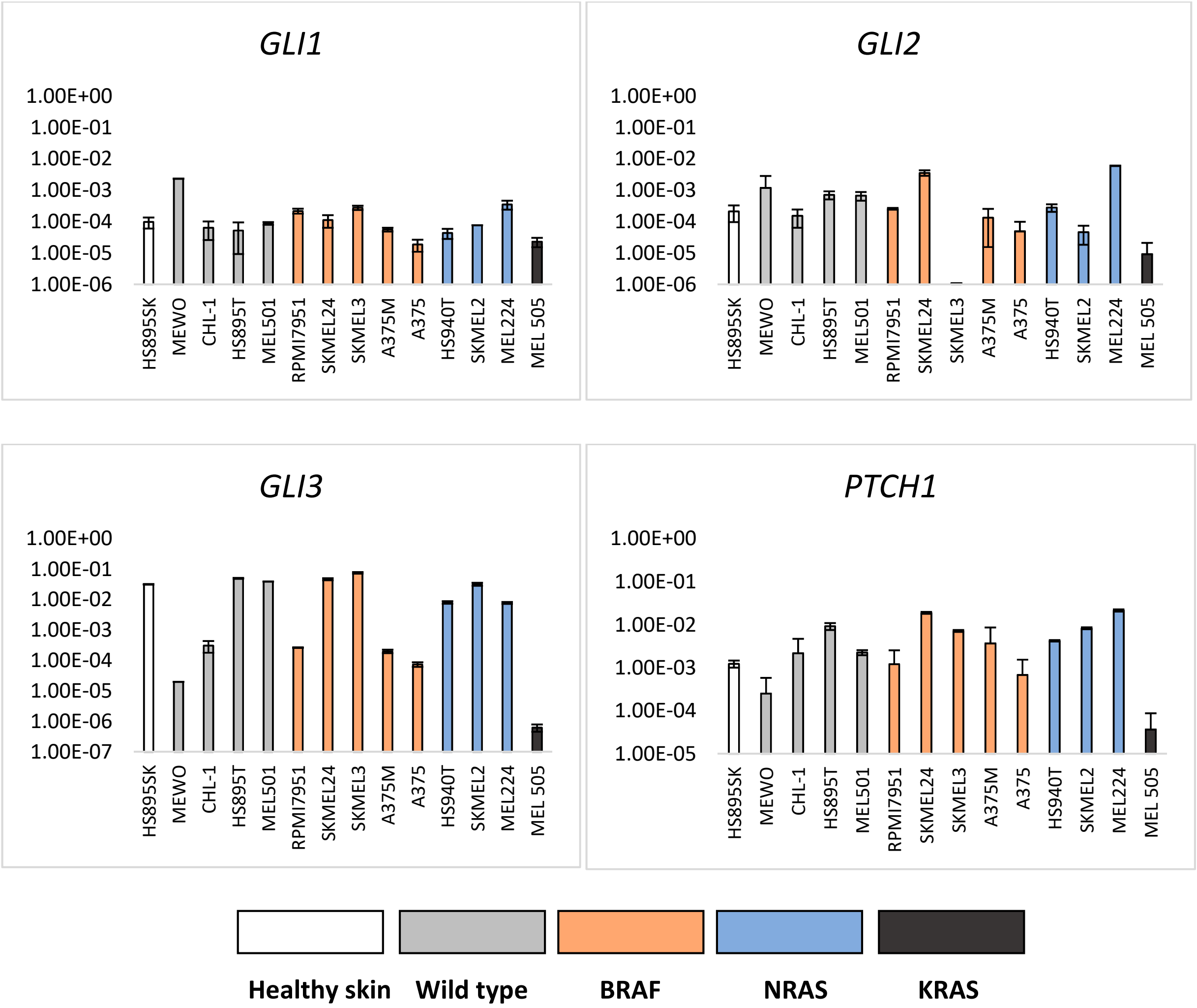

Supplementary Figure S1. HH-GLI pathway activity in melanoma cell lines. Average gene expression of *GLI1*, *GLI2*, *GLI3* and *PTCH1* in a panel of 14 melanoma cell lines. Expression is calculated as relative to the housekeeping gene *RPLP0,* and cell lines are grouped according to the mutational status of *BRAF*, *NRAS* and *KRAS* genes.

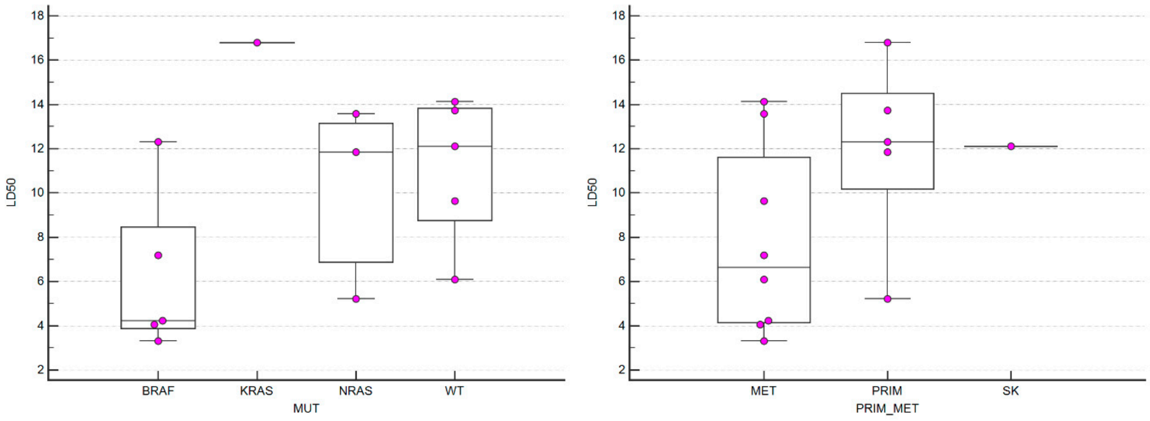

(**A**) (**B**)

Supplementary Figure S2. GANT61 IC50 values. IC50 values of inhibitor GANT61 for the tested melanoma cell lines according to the mutation status (**A**) or primary/metastatic status (**B**).

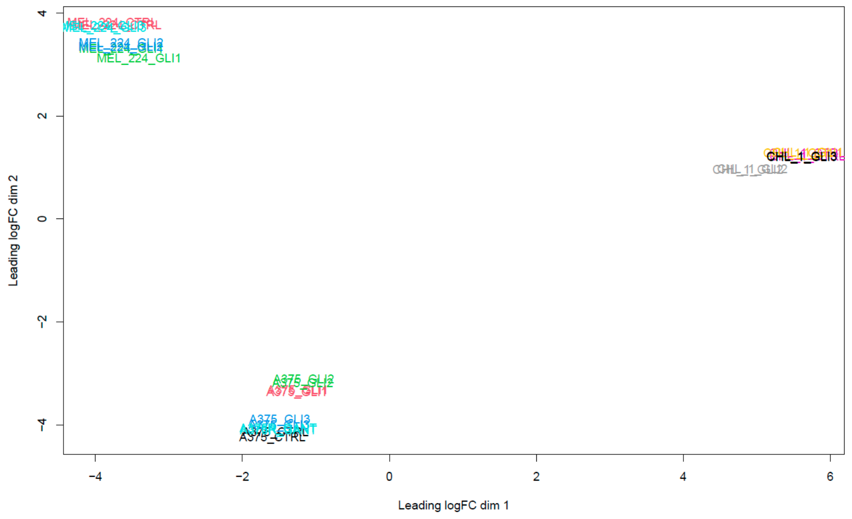

Supplementary Figure S3. MDS plot. MDS analysis for all samples used in RNA sequencing. Plot shows grouping according to three cell lines (CHL1, A375 and MEL224).

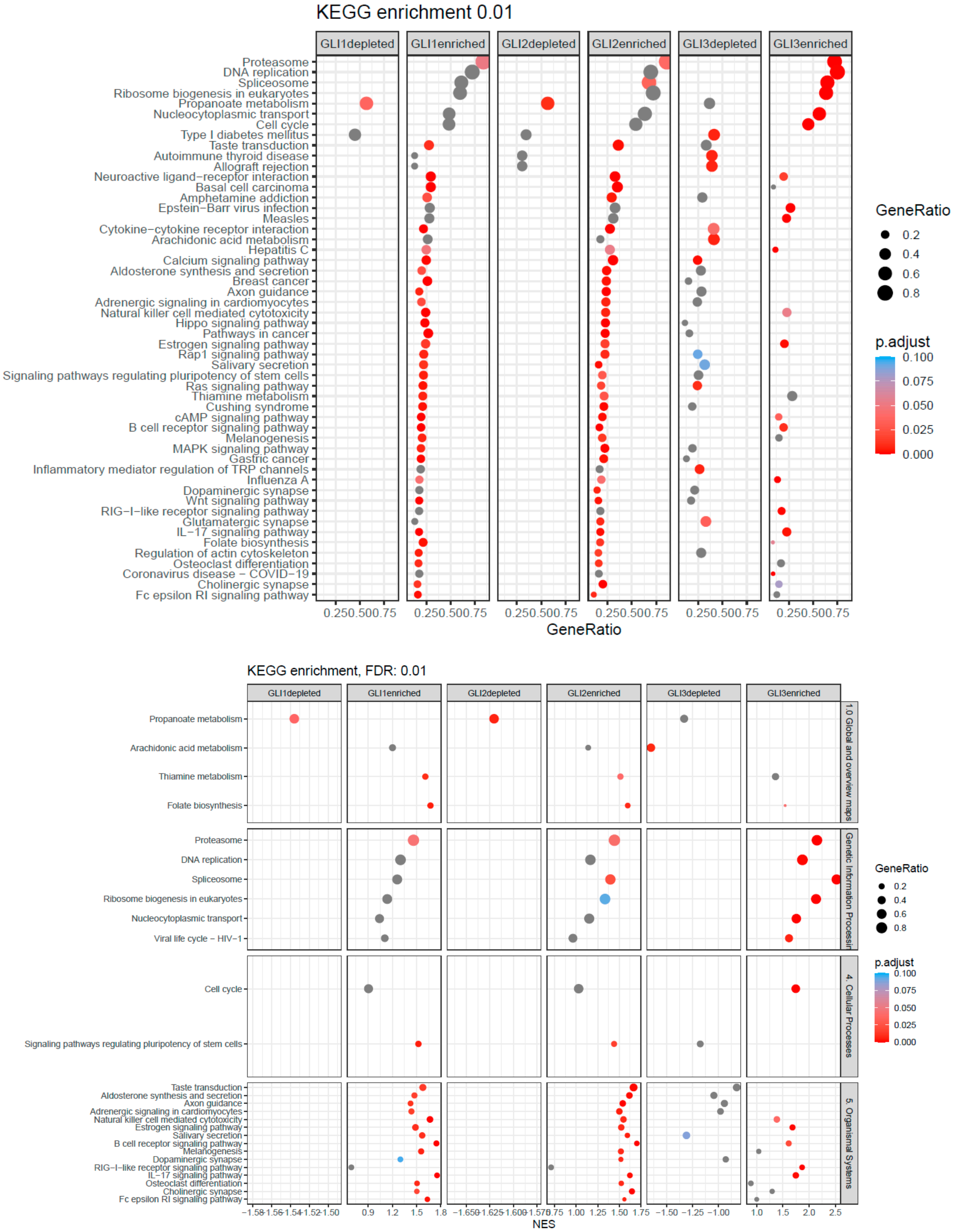

Supplementary Figure S4. KEGG pathway analysis. (A) all KEGG terms, (B) remaining KEGG categories not presented in Figure 2: Metabolism, Genetic information processing, Cellular processes and Organismal systems
