# Supplementary material for: RNA-seq and ChIP-seq Identification of Unique and Overlapping Targets of GLI Transcription Factors in Melanoma Cell Lines
Source: Cancers (Basel). 2022 Sep 19;14(18):4540. doi: 10.3390/cancers14184540 (PMC9497141; doi:10.3390/cancers14184540)
Supplement: Supplementary file 1 [file cancers-14-04540-s001.zip › Supplementary_Table_S1_primers.pdf]

Supplementary Table S1. List of primer sequences used for qPCR

KRT16	F: 5'-CTGAGACCTGGTTCCTGAGC-3'
	R: 5'-GTTCTCCAGGGATGCTTTCA-3'
KRT17	F: 5'-CGGAGACAGAGAACCGCTAC-3'
	R: 5'-TTCTTTCTTGTACTGAGTCAGGTG-3'
S100A9	F: 5'-CAGCTGGAACGCAACATAGA-3'
	R: 5'-TCAGCTGCTTGTCTGCATTT-3'
S100A7	F : 5'-TTCCCCAACTTCCTTAGTGC-3'
	R: 5'-CCCAGCAAGGACAGAAACTC-3'
GH1	F: 5'-CCTAGAGGAAGGCATCCAAA-3'
	R: 5'-GCAGCCCGTAGTTCTTGAGTAG-3'
SOX9	F: 5'-AGTACCCGCACTTGCACAAC-3'
	R: 5'-CGTTCTTCACCGACTTCCTC-3'
MRAS	F: 5'-CCTCATCGTCTACTCCGTCA-3'
	R: 5'-CATTGAGAGGTGGGTCCTTG-3'
BIRC7	F: 5'-CGTCTGGCCTCCTTCTATGA-3'
	R: 5'-CAGCAGCTGGGAGTGAGTCT-3'
IL1R2	F: 5'-TGGCACCTACGTCTGCACTA-3'
	R: 5'-TACCCCAGAGGTTGACAAGG-3'
EBI3	F: 5'-CAGCTTCGTGCCTTTCATAA-3'
	R: 5'-TCCCCGTAGTCTGTGAGGTC-3'
HES1	F: 5'-TGAGCCAGCTGAAAACACTG-3'
	R: 5'-CATTGATCTGGGTCATGCAG-3'
RET	F: 5'-CCACGCAAAGTGATGTATGG-3'
	R: 5'-CCGCAAGGTCCAAGTAGTCT-3'
SPRY2	F: 5'-CTCGGCCCAGAACGTGATT-3'
	R: 5'-GGCAAAAAGAGGGACATGACAC-3'
SAMMSON	F: 5'-GGAAAGAGGAAGCCATGAGG-3'
	R: 5'-ACTGCTGAGGACCCCAGTTT-3'
FLG	F: 5'-GGCAAATCCTGAAGAATCCA-3'
	R: 5'-TGCTTTCTGTGCTTGTGTCC-3'
RAB34	F: 5'-ATTACAAGGCCACCATTGGA-3'
	R: 5'-TGCAATGCATTTGAACCTCT-3'
CACNA2D2	F: 5'-TCATACGCGGCTGTACAGAT-3'
	R: 5'-ACAGGTCGATCTTCTTGGGG-3'
LAPTM5	F: 5'-TGCTAGCTCCTCCAAGTTCC-3'
	R: 5'-AAGGACAGTGATGAAGGCGA-3'
LY6D	F: 5'-CTCCCAGACGACATCAGAGA-3'
	R: 5'-GTGTTCGTGGTCTTGCAGAA-3'
RDH10	F: 5'-AGGCTGCTGAAAAGGATGGA-3'
	F: 5'-GTCACGATGTACATGAGGCG-3'
STK32C	F: 5'-CAGGACGAGGAGGACATGTT-3'
	R: 5'-GTGGCAATGTTGAAGTCGGT-3'
PTCH1	5' TCCTCGTGTGCGCTGTCTTCCTTC 3'
	5' CGTCAGAAAGGCCAAAGCAACGTGA 3'
GLI1	F: 5'-CTTGTGGTCCCCATGACTCT-3'
	R: 5'-GTTGGGGCTGGACATATCAC-3'
GLI2	5' GCCATCAAGACCGAGAGCTC 3'
	5' CGGCCCATGAGCAGGAATCC 3'
GLI3	5' CACTACCTCAAAGCGGGAAG 3'
	5' TGTTGGACTGTGTGCCATTT 3'
